# Changes in Self-Reported Empathy After a Basic Medical Communication Course Among Premedical Students: Single-Group Longitudinal Study

**DOI:** 10.2196/92215

**Published:** 2026-05-26

**Authors:** Seong Ju Jeon, Hyo Hyun Yoo

**Affiliations:** 1 College of Medicine, Jeonbuk National University, Jeonju-si, Jeonbuk-do, Republic of Korea; 2 Department of Medical Education, College of Medicine, Jeonbuk National University, 20, Geonji-ro, Deokjin-gu, Jeonju-si, Jeonbuk-do, 54907, Republic of Korea, 82 63-270-3881

**Keywords:** empathy, medical education, premedical students, physician-patient relationship, communication education

## Abstract

**Background:**

Empathy is essential to patient-centered care, yet erosion of empathy has been reported during medical training. Although educational interventions may support empathy development, longitudinal evidence on whether these changes persist remains limited, particularly in non-Western settings.

**Objective:**

This study examined changes in overall, emotional, and cognitive self-reported empathy scores and confidence in applying empathy at baseline, immediately after, and 6 months after a basic medical communication course among Korean premedical students. It also explored whether trajectories in self-reported empathy scores differed by gender.

**Methods:**

In this single-group longitudinal study, 82.1% (119/145) of second-year premedical students completed surveys at all 3 time points. The required 15-week course included 12 instructional hours on medical communication theory, nonviolent communication, and peer clinical interview role-play. No formal communication- or empathy-focused instruction was provided during follow-up. Self-reported empathy scores were measured using the Korean student version of the Jefferson Scale of Empathy, and 2 additional items assessed confidence in applying empathy in daily life and in physician-patient relationships. Repeated-measures and mixed-design repeated-measures ANOVAs were used.

**Results:**

Overall self-reported empathy scores changed significantly over time (*F*_2,236_=13.41; *P*<.001), increasing from baseline (mean 5.30, SD 0.57) to immediately after the course (mean 5.55, SD 0.62) and remaining above baseline at 6 months (mean 5.48, SD 0.60). Emotional empathy scores increased from baseline to immediately after the course and remained above baseline at 6 months (mean 5.43, SD 0.58; mean 5.66, SD 0.63; and mean 5.59, SD 0.61, respectively; *F*_2,236_=10.67; *P*<.001), as did cognitive empathy scores (mean 4.10, SD 1.18; mean 4.50, SD 1.30; and mean 4.51, SD 1.24, respectively; *F*_2,236_=8.80; *P*<.001). Female students had higher overall empathy scores across assessments than male students (*F*_1,117_=6.09; *P*=.02), but time-by-gender interactions were not significant for overall, emotional, or cognitive empathy (*F*_2,234_=0.33 and *P*=.72, *F*_2,234_=0.32 and *P*=.73, and *F*_2,234_=0.12 and *P*=.89, respectively). Confidence in applying empathy in daily life increased from baseline to immediately after the course and remained higher at 6 months (mean 5.02, SD 1.04; mean 5.26, SD 0.97; and mean 5.26, SD 0.82, respectively; *F*_2,236_=5.18; *P*=.006). Confidence in applying empathy in physician-patient relationships showed a significant overall time effect (mean 4.91, SD 1.09; mean 5.13, SD 1.04; and mean 5.13, SD 0.97, respectively; *F*_2,236_=3.46; *P*=.03), but Bonferroni-adjusted pairwise comparisons were not significant (baseline vs immediately after the course, adjusted *P*=.07; baseline vs 6-month follow-up, adjusted *P*=.06; and immediately after the course vs 6-month follow-up, adjusted *P*>.99).

**Conclusions:**

Participation in a basic medical communication course was associated with higher self-reported empathy scores and greater confidence in applying empathy in daily life, with gains maintained at 6 months; empathy trajectories did not differ significantly by gender. Controlled studies are needed to determine the independent and sustained effects of the course.

## Introduction

Empathy is the ability to understand and share another person’s feelings from that person’s perspective and comprises cognitive and emotional dimensions [1]. In medicine, empathy is essential for forming therapeutic relationships, enabling physicians to better understand patients’ concerns and build trust [2,3]. Empathetic physician-patient relationships have been associated with better clinical outcomes, patient satisfaction, and treatment adherence [2,3]. Research on perceptions of good physicians also suggests that patients value physicians who demonstrate both clinical competence and empathy [4]. Together, these findings support the view that empathy is a core construct in medical education and assessment, underscoring the need to foster empathy-related competence early in medical training [4,5].

Despite its importance, empathy decline during medical training has been widely reported and is often described as an “erosion of empathy” [6-8]. Empathy levels have been reported to vary by gender and educational experience [9,10]. These findings suggest that empathy should not be treated as a fixed trait but as a construct that may change across medical education. Because empathy scores during medical school have also been linked to later empathic behavior in residency training [11], efforts to support empathy may be valuable from the early stages of training, before students enter more intensive clinical environments [6,12,13].

Although empathy may have personal or dispositional components, educational reinforcement is considered important for developing and sustaining empathy-related attitudes and behaviors [14]. While several international programs have aimed to enhance medical students’ empathy [15,16], longitudinal evidence on the durability of such interventions remains limited [17], particularly in Korean medical education. Previous Korean studies have primarily examined differences by gender, academic year, and educational system or have focused on developing empathy measurement tools [18,19]. However, longitudinal evidence on changes in empathy after structured empathy- or communication-focused educational programs in Korean premedical settings remains limited. In addition to self-reported empathy, students’ confidence in applying empathy may provide an exploratory indicator of their perceived readiness to use empathic communication.

Accordingly, this single-group longitudinal study aimed to examine changes in overall, emotional, and cognitive self-reported empathy scores and confidence in applying empathy among Korean premedical students at baseline, immediately after, and 6 months after a required basic medical communication course. It also explored whether empathy trajectories differed by gender.

## Methods

### Study Design and Setting

This study used a single-group longitudinal design to examine changes in self-reported empathy and confidence in applying empathy at 3 time points: before the course, immediately after the final examination, and 6 months after course completion. The study was conducted at Jeonbuk National University College of Medicine. The basic medical communication course was delivered during the second year of premedical education as a required face-to-face course.

### Participants

Participants were second-year premedical students enrolled in the basic medical communication course. Of 145 eligible students, 119 (82.1%) completed surveys at all 3 assessment points and were included in the longitudinal analyses. The final sample included 63% (75/119) male students and 37% (44/119) female students.

### Survey Administration

Surveys were administered online using Google Forms at 3 time points: before the first class, immediately after the final examination, and 6 months after course completion. At each time point, the survey link was distributed only to students enrolled in the basic medical communication course.

The first page of the online survey provided information about the study purpose and procedures, the voluntary nature of participation, and confidentiality. Students proceeded to the survey only after indicating informed consent by checking the consent item.

Students used an anonymous code to allow for longitudinal matching of responses across the 3 survey time points. Survey participation was voluntary and unrelated to attendance, examination scores, final course grades, or academic standing.

### Overview of the Educational Program

The educational program evaluated in this study was the basic medical communication course, a required face-to-face course delivered during the second year of premedical education. Within the broader medical communication curriculum at our institution, this course precedes three subsequent required courses—Medical Communication 1, Medical Communication 2, and Medical Communication 3—as well as simulated patient interview training in another required course, Introduction to Clinical Medicine. No formal communication- or empathy-focused course was offered between the immediately postcourse assessment and the 6-month follow-up for this cohort.

**Figure 1.**
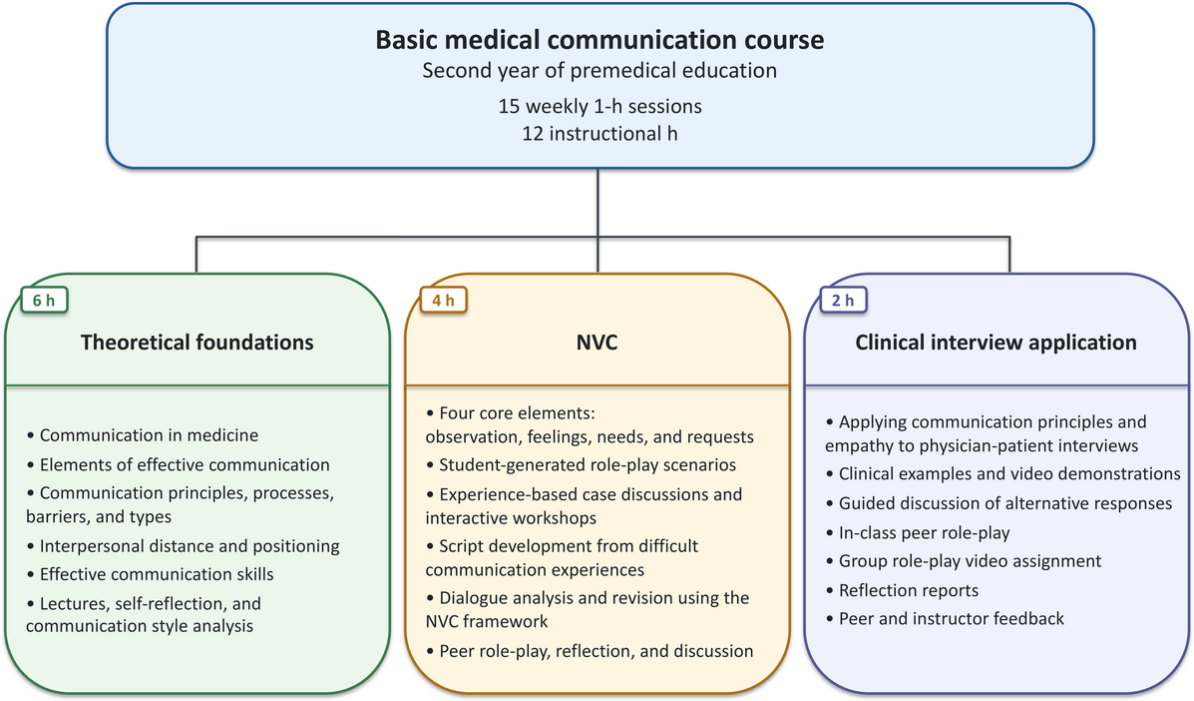
Overview of the basic medical communication course. NVC: nonviolent communication.

The course comprised 15 weekly 1-hour sessions. Of these, 12 hours were instructional sessions, whereas 3 hours were allocated to orientation, the midterm examination, and the final examination. The 12 instructional hours were organized into 3 components: theoretical foundations of medical communication, nonviolent communication (NVC), and application of communication principles to clinical interview contexts. An overview of the course is shown in Figure 1.

The theoretical component comprised 6 hours and was delivered mainly through face-to-face lectures. This component covered the importance of communication in medicine, elements of effective communication, basic principles and processes of communication, barriers and types of communication, interpersonal distance and positioning, and effective communication skills. Learning activities included lectures, self-reflection, and communication style analysis.

The NVC component comprised 4 hours and included student-generated role-play scenarios, experience-based case discussions, and interactive workshops. Students were introduced to the 4 core elements of NVC: observation, feelings, needs, and requests [20]. In this component, students developed scripts based on recent experiences in which communication had been ineffective or difficult, analyzed the communication difficulty using the NVC framework, revised the dialogue, and practiced the revised conversation through peer role-play. These activities were followed by reflection and discussion.

The clinical interview application component comprised 2 hours and focused on connecting communication principles and empathy to physician-patient interview contexts. This component included lectures, clinical examples, video demonstrations of physician-patient interviews, guided discussion, and role-play scenarios. During class, students discussed possible responses to specific clinical communication situations and compared alternative responses in terms of contextual appropriateness. Students then practiced in-class peer role-play based on the clinical examples. As an additional assignment, students worked in groups of 7 to 10 to develop their own physician-patient communication scenario and produce a role-play video. Students submitted reflection reports with the video assignment. The submitted videos were reviewed in class, followed by peer and instructor feedback.

### Measures

Self-reported empathy was assessed using the Korean student version of the Jefferson Scale of Empathy (S-JSE-K) adapted from the Jefferson Scale of Empathy [21] and translated and validated for Korean medical students [19,22]. The S-JSE-K consists of 18 items rated on a 7-point Likert scale and includes 2 domains: emotional empathy (16 items) and cognitive empathy (2 items). Scale and subscale scores were calculated as item means, with higher scores indicating greater self-reported empathy. Internal consistency in this sample was good for the total scale (Cronbach α=0.86) and emotional empathy (Cronbach α=0.88) and acceptable for cognitive empathy (Cronbach α=0.76).

In addition, 2 single items assessed confidence in applying empathy in daily life and in physician-patient relationships. These items were rated on the same 7-point Likert scale, with higher scores indicating greater perceived confidence.

### Statistical Analysis

Means and SDs were calculated for overall empathy, emotional empathy, cognitive empathy, and the 2 confidence items at each assessment point. Repeated-measures ANOVA was used to examine within-subject changes over time, and Bonferroni-adjusted pairwise comparisons were conducted post hoc among baseline, immediately postcourse, and 6-month follow-up scores. Sphericity was assessed using the Mauchly test. To examine whether empathy trajectories differed by gender, mixed-design repeated-measures ANOVA was conducted with time as the within-subject factor and gender as the between-subject factor. Partial η^2^ was reported as an effect size. All analyses were performed using PASW Statistics (version 18.0; SPSS Inc). Two-sided *P* values of less than .05 were considered statistically significant.

### Ethical Considerations

The institutional review board of Jeonbuk National University approved this study (2020-02-002-002). All participants received information about the study purpose and procedures before survey completion and provided informed consent. Participation was voluntary and did not affect course grades or academic standing. No financial compensation, course credit, or other incentives were provided for survey participation. Survey data were analyzed in deidentified form. The study was conducted in accordance with the principles of the Declaration of Helsinki.

## Results

### Changes in Self-Reported Empathy

Repeated-measures ANOVA showed a significant effect of time on overall empathy (*F*_2,236_=13.41; *P*<.001; partial η^2^=0.10). Bonferroni-adjusted pairwise comparisons showed that overall empathy was higher immediately after the course than at baseline (adjusted *P*<.001) and remained higher at the 6-month follow-up than at baseline (adjusted *P*<.001). The immediately postcourse and 6-month follow-up scores did not differ significantly (adjusted *P*=.65).

**Figure 2.**
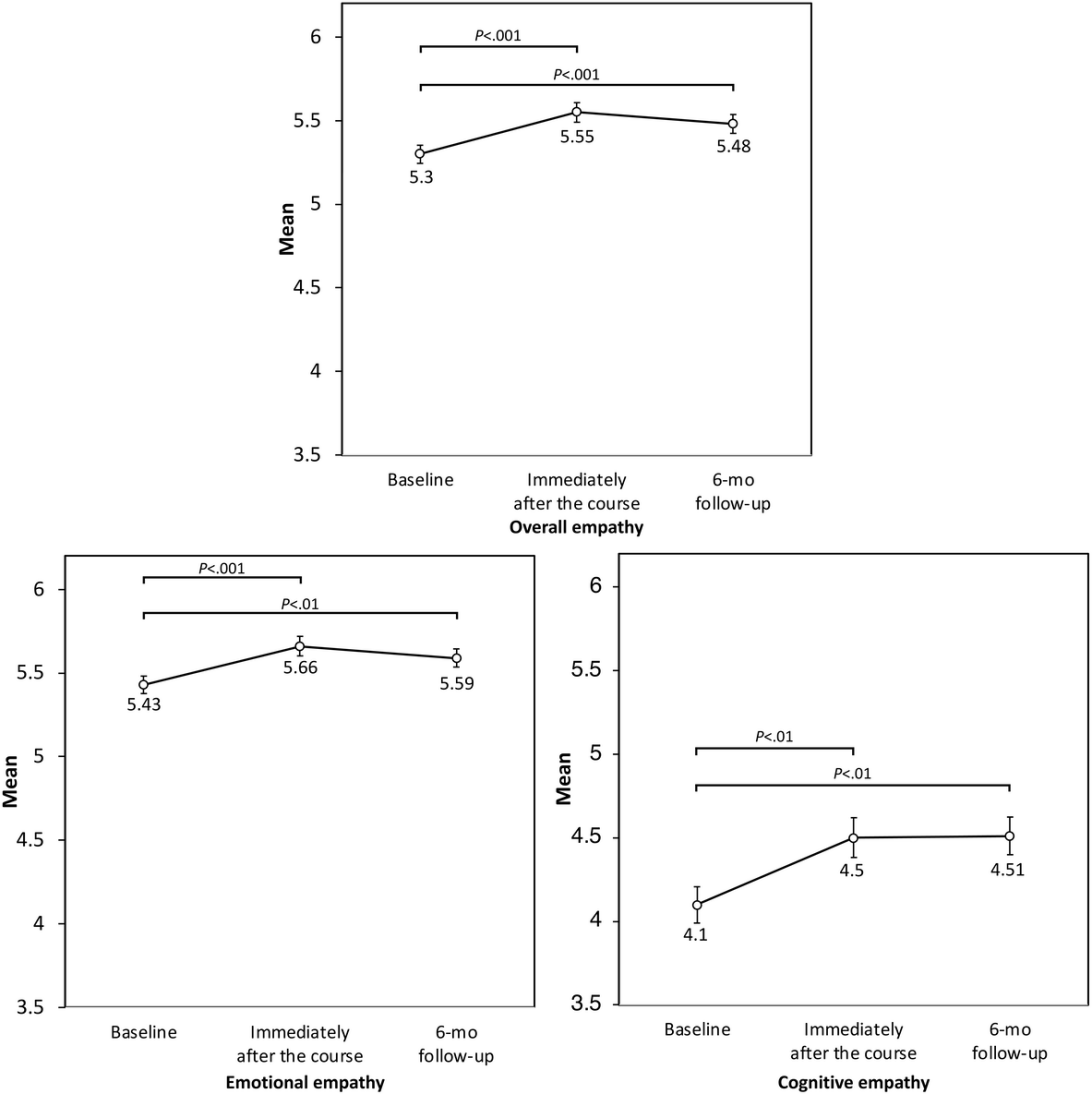
Changes in overall, emotional, and cognitive empathy scores across time points. Error bars indicate SEs.

Significant time effects were also observed for emotional empathy (*F*_2,236_=10.67; *P*<.001; partial η^2^=0.08) and cognitive empathy (*F*_2,236_=8.80; *P*<.001; partial η^2^=0.07). Emotional empathy was higher immediately after the course than at baseline (adjusted *P*<.001) and higher at follow-up than at baseline (adjusted *P*=.007), whereas the immediately postcourse and follow-up scores did not differ significantly (adjusted *P*=.51). Cognitive empathy was higher immediately after the course than at baseline (adjusted *P*=.002) and higher at follow-up than at baseline (adjusted *P*=.001), whereas the immediately postcourse and follow-up scores did not differ significantly (adjusted *P*>.99). Detailed Bonferroni-adjusted pairwise comparisons can be found in Table S1 in Multimedia Appendix 1. These results are summarized in Table 1 and illustrated in Figure 2.

**Table 1.** Changes in self-reported empathy across time points^a^.

	Baseline, mean (SD)	Immediately after the course, mean (SD)	6-mo follow-up, mean (SD)	*F* test (*df*)	*P* value	Partial η^2^
Overall empathy	5.30 (0.57)	5.55 (0.62)	5.48 (0.60)	13.41 (2,236)	<.001	0.10
Emotional empathy	5.43 (0.58)	5.66 (0.63)	5.59 (0.61)	10.67 (2,236)	<.001	0.08
Cognitive empathy	4.10 (1.18)	4.50 (1.30)	4.51 (1.24)	8.80 (2,236)	<.001	0.07

a S-JSE-K scores were calculated as item means and range from 1 to 7, with higher scores indicating greater self-reported empathy.

### Gender Differences in Empathy Trajectories

**Figure 3.**
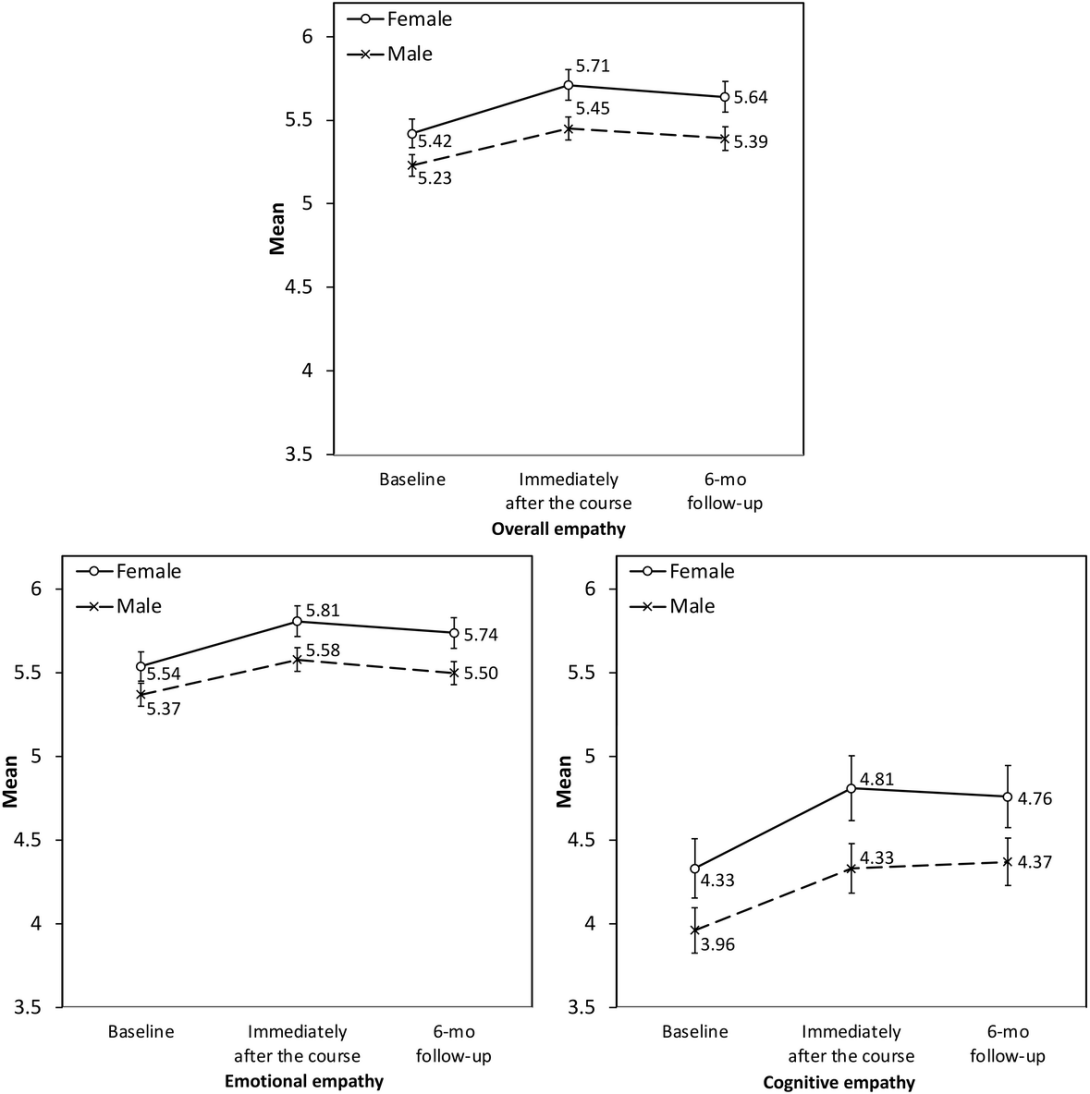
Changes in overall, emotional, and cognitive empathy scores across time points by gender. Error bars indicate SEs.

At baseline, female students reported higher overall empathy scores than male students (mean 5.42, SD 0.58 vs mean 5.23, SD 0.56), and this pattern remained at the immediately postcourse assessment (mean 5.71, SD 0.56 vs mean 5.45, SD 0.63) and 6-month follow-up (mean 5.64, SD 0.52 vs mean 5.39, SD 0.64). Similar patterns were observed for emotional and cognitive empathy; gender-stratified means and SDs are provided in Table S2 in Multimedia Appendix 1. Mixed-design repeated-measures ANOVA showed no significant time-by-gender interaction for overall empathy (*F*_2,234_=0.33; *P*=.72), emotional empathy (*F*_2,234_=0.32; *P*=.73), or cognitive empathy (*F*_2,234_=0.12; *P*=.89), suggesting no evidence that empathy trajectories differed by gender. Female students had higher mean scores across the 3 assessment points for overall empathy (*F*_1,117_=6.09; *P*=.02; partial η^2^=0.05), emotional empathy (*F*_1,117_=4.91; *P*=.03; partial η^2^=0.04), and cognitive empathy (*F*_1,117_=4.78; *P*=.03; partial η^2^=0.04). These results are summarized in Table 2 and illustrated in Figure 3.

**Table 2.** Mixed-design repeated-measures ANOVA results for gender differences in empathy over time.

Effect	*F* test (*df*)	*P* value	Partial η^2^
Overall empathy			
Time	13.48 (2,234)	<.001	0.10
Time × gender	0.33 (2,234)	.72	<0.01
Gender main effect	6.09 (1,117)	.02	0.05
Emotional empathy			
Time	10.77 (2,234)	<.001	0.08
Time × gender	0.32 (2,234)	.73	<0.01
Gender main effect	4.91 (1,117)	.03	0.04
Cognitive empathy			
Time	8.49 (2,234)	<.001	0.07
Time × gender	0.12 (2,234)	.89	<0.01
Gender main effect	4.78 (1,117)	.03	0.04

### Changes in Confidence in Applying Empathy

Repeated-measures ANOVA showed a significant effect of time on confidence in applying empathy in daily life (*F*_2,236_=5.18; *P*=.006; partial η^2^=0.04). Bonferroni-adjusted pairwise comparisons showed that confidence in daily life was significantly higher immediately after the course than at baseline (adjusted *P*=.02) and remained significantly higher at the 6-month follow-up than at baseline (adjusted *P*=.02). The immediately postcourse and follow-up scores did not differ significantly (adjusted *P*>.99).

**Figure 4.**
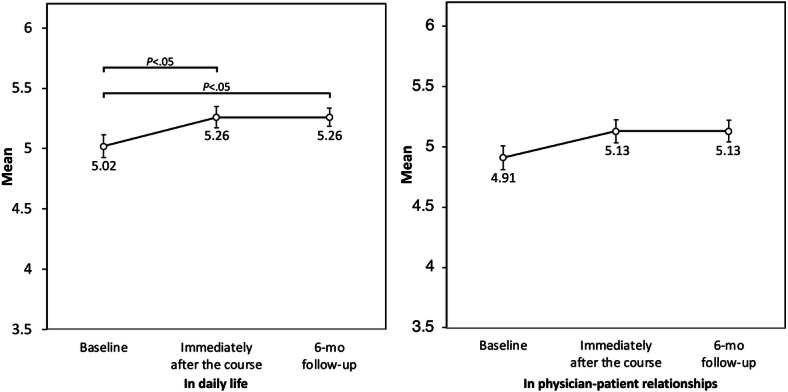
Changes in confidence in applying empathy in daily life and in physician-patient relationships across time points. Error bars indicate SEs.

Confidence in applying empathy in physician-patient relationships also showed a significant overall effect of time (*F*_2,236_=3.46; *P*=.03; partial η^2^=0.03). However, Bonferroni-adjusted pairwise comparisons did not reach statistical significance for the comparison from baseline to immediately after the course (adjusted *P*=.07) or from baseline to follow-up (adjusted *P*=.06). The immediately postcourse and follow-up scores did not differ significantly (adjusted *P*>.99). These findings are summarized in Table 3 and illustrated in Figure 4. Detailed Bonferroni-adjusted pairwise comparisons are provided in Table S3 in Multimedia Appendix 1.

**Table 3.** Changes in confidence in applying empathy across time points^a^.

	Baseline, mean (SD)	Immediately after the course, mean (SD)	6-mo follow-up, mean (SD)	*F* test (*df*)	*P* value	Partial η^2^
Confidence in applying empathy in daily life	5.02 (1.04)	5.26 (0.97)	5.26 (0.82)	5.18 (2,236)	.006	0.04
Confidence in applying empathy in physician-patient relationships	4.91 (1.09)	5.13 (1.04)	5.13 (0.97)	3.46 (2,236)	.03	0.03

a Confidence scores range from 1 to 7, with higher scores indicating greater perceived confidence.

## Discussion

### Principal Results

In this single-group longitudinal study, self-reported empathy scores increased during the course period and were maintained at 6 months, with similar patterns for emotional and cognitive empathy scores. Female students reported higher overall empathy scores than male students, but no significant time-by-gender interaction was observed, indicating similar longitudinal trajectories between male and female students. Confidence in applying empathy in daily life increased and remained above baseline at follow-up. Confidence in applying empathy in physician-patient relationships showed a significant overall time effect, although Bonferroni-adjusted pairwise comparisons were not statistically significant.

### Comparison With Prior Work

The finding that self-reported empathy scores were higher after the course is consistent with prior work suggesting that empathy-related attitudes and communication skills can be supported through structured educational approaches [15,16,23-26]. A recent meta-analysis reported that empathy-focused interventions in medical students and physicians had moderate effects on both cognitive and affective empathy [27].

The findings of this study are also relevant to the literature on empathy trajectories during medical training. Several studies have reported decreases in empathy during medical school, especially during later clinical training [7,8,12,13]. However, evidence is not entirely consistent across settings. A longitudinal study in Singapore found no significant overall decline in empathy across medical education and suggested that curricular and sociocultural context may influence empathy trajectories [28]. In the Korean context, prior studies have focused mainly on empathy correlates, measurement, curricular attitudes, or clerkship-associated change rather than on longitudinal intervention studies in premedical students [18,19,29,30]. In this context, this study adds preliminary longitudinal evidence from a Korean premedical setting. That empathy scores were still elevated at 6 months is encouraging, although the modest attenuation after the immediately postcourse assessment suggests that a single short course may be insufficient to support durable long-term development without later reinforcement [17].

Because this study did not include a control group, the findings should be interpreted as longitudinal changes associated with course participation rather than definitive evidence of course effectiveness. Although no formal communication- or empathy-focused instruction took place during follow-up, other unmeasured experiences may also have influenced the outcomes.

### Emotional and Cognitive Empathy

Both emotional and cognitive empathy scores showed significant time effects and remained above baseline at the 6-month follow-up. Apparent differences between the 2 subscales should be interpreted cautiously. Although the raw mean increase was numerically larger for cognitive empathy, this should not be taken as evidence that the course had a stronger influence on cognitive perspective taking. The cognitive empathy subscale of the S-JSE-K contains only 2 items compared with 16 items in the emotional empathy subscale, and cognitive empathy scores also had a lower baseline mean and greater variability. Together, these features may have made the cognitive empathy score less precise and more sensitive to item-level variation.

The parallel increases in emotional and cognitive empathy may reflect the course’s broader combination of conceptual learning and practice-based communication activities rather than any single instructional component [15,16,23,25-27]. Because the intervention was evaluated as a whole, this study cannot determine which specific components were most closely associated with changes in each empathy domain. Future studies should use more balanced multidimensional empathy measures, component-level process data, and observational outcomes to clarify how communication-focused education is associated with changes in emotional empathy, cognitive empathy, and empathic behavior.

### Gender Differences

Female students reported higher empathy scores than male students across the 3 assessment points. This finding is consistent with those of previous studies reporting higher empathy scores among female medical students [31-35]. However, the absence of significant time-by-gender interactions indicates that the pattern of change over time was similar between male and female students. Thus, although female students had higher mean scores across assessments, the course-associated longitudinal trajectory of empathy scores did not appear to differ by gender.

Several explanations have been proposed for gender differences in self-reported empathy scores, including emotional socialization, responsiveness to interpersonal cues, communication expectations, and self-report tendencies [33,35,36]. These factors may be particularly relevant when empathy is measured using self-report instruments because responses may reflect not only empathic orientation but also gendered expectations about emotional expressiveness and interpersonal sensitivity [26]. In the Korean premedical context, these findings should be interpreted in light of broader sociocultural patterns in empathy and emotional expression [37,38]. Findings from Korean medical education studies have not been fully consistent, with at least one Korean study reporting an atypical gender pattern in empathy scores before clerkship [30]. Therefore, the higher mean scores among female students in this study should be interpreted as a descriptive group difference rather than evidence of fixed gender-based differences in empathic capacity. Future research should examine how gender, cultural context, self-report tendencies, and prior communication experiences interact to shape empathy score trajectories in Korean medical education.

### Confidence in Applying Empathy

Confidence in applying empathy in daily life increased significantly from baseline to immediately after the course and remained significantly higher than baseline at follow-up. Confidence in applying empathy in physician-patient relationships also showed a significant overall time effect and remained numerically unchanged from immediately after the course to follow-up, although Bonferroni-adjusted pairwise comparisons from baseline were not statistically significant.

These findings suggest that students’ perceived readiness to apply empathy may be more stable than their self-reported empathy scores over the 6-month period. Empathy scores reflect students’ self-reported empathic orientation, whereas confidence items reflect perceived ability or readiness to use empathy in specific contexts. The slight decline in empathy scores at follow-up may reflect natural fluctuation or the challenge of sustaining empathy-related attitudes over time. In contrast, the relatively stable confidence scores may indicate that students retained a practical sense of how to apply empathic communication after the course.

However, confidence should not be equated with demonstrated empathic competence [39]. The confidence outcomes were measured using 2 items and were exploratory in nature. Future studies should examine whether confidence in applying empathy is associated with observed communication behaviors, standardized patient ratings, observer ratings, faculty assessments, or patient-reported empathy measures.

### Implications for Medical Education

These findings suggest the potential educational value of introducing empathy and communication training during the premedical phase. Early exposure may help students acquire a shared language for patient-centered communication and begin linking empathy to observable communication behaviors. At the same time, the temporal pattern suggests that an introductory course should be viewed as a starting point rather than a complete solution. Most score changes occurred during the course itself, and the 6-month pattern was more consistent with short-term maintenance than with continued growth.

Although this pattern does not establish causality, it supports a longitudinal, spiral model in which early communication training is reinforced through later experiential and reflective opportunities [25,26,40,41]. Such integration may help students move beyond self-reported empathic orientation toward more stable empathic communication in authentic clinical settings.

### Limitations

This study has several limitations. First, it was conducted at a single institution with Korean premedical students, which may limit generalizability to other institutions, countries, or stages of medical training. Second, the single-group design without a comparison group limits causal inference, and other unmeasured experiences during follow-up may have influenced the outcomes. Third, all outcomes were based on self-report. Self-reported empathy and confidence may not directly correspond to empathic behavior in authentic clinical encounters [26,39]. Future studies should include behavioral assessments, standardized patient ratings, peer or faculty ratings, or patient-reported measures. Fourth, the cognitive empathy subscale of the S-JSE-K contains only 2 items, which may limit its sensitivity to detecting changes in cognitive empathy. Fifth, the 2 confidence items were single-item exploratory measures and were not validated as independent subscales. Finally, the follow-up period was limited to 6 months. Longer longitudinal studies are needed to examine whether course-associated changes persist into clinical training and whether they are reflected in observed physician-patient communication.

### Conclusions

In this single-group longitudinal study, participation in a basic medical communication course was associated with higher self-reported empathy scores and greater confidence in applying empathy in daily life at 6 months among Korean premedical students. Controlled multisite studies using behavioral outcomes are needed to determine whether these early gains translate into sustained empathic communication during later clinical training.

## Supplementary material

10.2196/92215 Multimedia Appendix 1 Bonferroni-adjusted pairwise comparisons for self-reported empathy and confidence outcomes and gender-stratified descriptive statistics for self-reported empathy.

## Abbreviations

NVC: nonviolent communication
S-JSE-K: Korean student version of the Jefferson Scale of Empathy
