# Supplementary material for: Changes in Self-Reported Empathy After a Basic Medical Communication Course Among Premedical Students: Single-Group Longitudinal Study
Source: JMIR Med Educ. 2026 May 26;12:e92215. doi: 10.2196/92215 (PMC13211606; doi:10.2196/92215)
Supplement: Multimedia Appendix 1 [file mededu-v12-e92215-s001.pdf]

## Bonferroni-adjusted pairwise comparisons for self-reported empathy and confidence outcomes and gender-stratified descriptive statistics for self-reported empathy.

### Table S1. Bonferroni-adjusted pairwise comparisons for self-reported empathy scores

| Outcome | Pairwise comparison | Mean difference[a] | Bonferroni-adjusted *P* value[b] |
|---|---|---|---|
| Overall empathy | Immediate postcourse vs baseline | 0.25 | <.001 |
| | 6-month follow-up vs baseline | 0.18 | <.001 |
| | 6-month follow-up vs immediate postcourse | -0.07 | .65 |
| Emotional empathy | Immediate postcourse vs baseline | 0.23 | <.001 |
| | 6-month follow-up vs baseline | 0.16 | .007 |
| | 6-month follow-up vs immediate postcourse | -0.07 | .51 |
| Cognitive empathy | Immediate postcourse vs baseline | 0.41 | .002 |
| | 6-month follow-up vs baseline | 0.42 | .001 |
| | 6-month follow-up vs immediate postcourse | 0.01 | >.99 |

Note. Scores range from 1 to 7, with higher scores indicating greater self-reported empathy. [a]Mean differences were calculated as the later assessment score minus the earlier assessment score using unrounded mean scores. [b]*P* values were adjusted using the Bonferroni method within each outcome.

### Table S2. Gender-stratified descriptive statistics for self-reported empathy scores

| Outcome | Gender | Baseline, mean (SD) | Immediate postcourse, mean (SD) | 6-month follow-up, mean (SD) |
|---|---|---|---|---|
| Overall empathy | Male (n=75) | 5.23 (0.56) | 5.45 (0.63) | 5.39 (0.64) |
| | Female (n=44) | 5.42 (0.58) | 5.71 (0.56) | 5.64 (0.52) |
| Emotional empathy | Male (n=75) | 5.37 (0.57) | 5.58 (0.64) | 5.50 (0.65) |
| | Female (n=44) | 5.54 (0.60) | 5.81 (0.57) | 5.74 (0.52) |
| Cognitive empathy | Male (n=75) | 3.96 (1.13) | 4.33 (1.28) | 4.37 (1.27) |
| | Female (n=44) | 4.33 (1.25) | 4.81 (1.29) | 4.76 (1.16) |

Note. Scores range from 1 to 7, with higher scores indicating greater self-reported empathy.

**Table S3. Bonferroni-adjusted pairwise comparisons for confidence in applying empathy**

| Outcome | Pairwise comparison | Mean difference[a] | Bonferroni-adjusted *P* value[b] |
|---|---|---|---|
| Confidence in applying empathy in daily life | Immediate postcourse vs baseline | 0.24 | .02 |
| | 6-month follow-up vs baseline | 0.24 | .02 |
| | 6-month follow-up vs immediate postcourse | 0.00 | >.99 |
| Confidence in applying empathy in doctor-patient relationships | Immediate postcourse vs baseline | 0.23 | .07 |
| | 6-month follow-up vs baseline | 0.23 | .06 |
| | 6-month follow-up vs immediate postcourse | 0.00 | >.99 |

Note. Scores range from 1 to 7, with higher scores indicating greater perceived confidence. [a]Mean differences were calculated as the later assessment score minus the earlier assessment score using unrounded mean scores. [b]*P* values were adjusted using the Bonferroni method within each outcome.
